# Cardiac Resynchronization Therapy Upgrade in a Patient with Dextrocardia and Situs Inversus Totalis, Facilitated by Coronary Sinus Cannulation with Electrophysiology Catheters from Both Femoral and Axillary Venous Approaches

**DOI:** 10.19102/icrm.2018.090401

**Published:** 2018-04-15

**Authors:** Mazda Motallebi, Narges Feizabadi

**Affiliations:** ^1^Cardiovascular and Electrophysiology Services, CareMore Health Plan, Los Angeles, CA, USA; ^2^Cardiac Electrophysiology Laboratory, Lakewood Regional Medical Center, Lakewood, CA, USA

**Keywords:** Cardiac resynchronization therapy, coronary sinus, dextrocardia, left ventricular lead, pacemaker

## Abstract

Cardiac resynchronization therapy in patients with dextrocardia with situs inversus totalis can be technically challenging. There are few case reports of cardiac resynchronization therapy implantation in these individuals. Here, we describe a procedure of cardiac resynchronization therapy upgrade in a patient with dextrocardia and situs inversus totalis facilitated by coronary sinus cannulation from both the femoral and axillary venous approaches.

## Introduction

Cardiac resynchronization therapy (CRT) in patients with dextrocardia situs inversus totalis (DX-SIT) can be technically challenging. To date, there is only a small number of case reports of this procedure in the literature.^1–4^ DX-SIT is a rare condition in which major visceral organs including the heart are located at a mirror image position of their normal placement that affects one to two per 10,000 live births annually.^5^ Less than 10% of individuals with DX-SIT have associated congenital cardiac defects^6^; however, in those who do, anomalous venous returns are common. In patients with DX-SIT, the normal superior vena cava (SVC) is located at the left side of the spine as the mirror image of normal anatomy. DX-SIT can be associated with persistent right SVC, which may complicate or, in some cases, facilitate transvenous lead implantation.^6^

Congenital heart block may coexist with dextrocardia.^7^ There are case reports of early to mid-adulthood presentations of complete heart block in association with dextrocardia.^8,9^ This phenomenon could possibly be due to idiopathic degeneration of the conduction system or delayed requirement for cardiac pacing associated with congenital heart block.

## Case presentation

**Figure 1:**
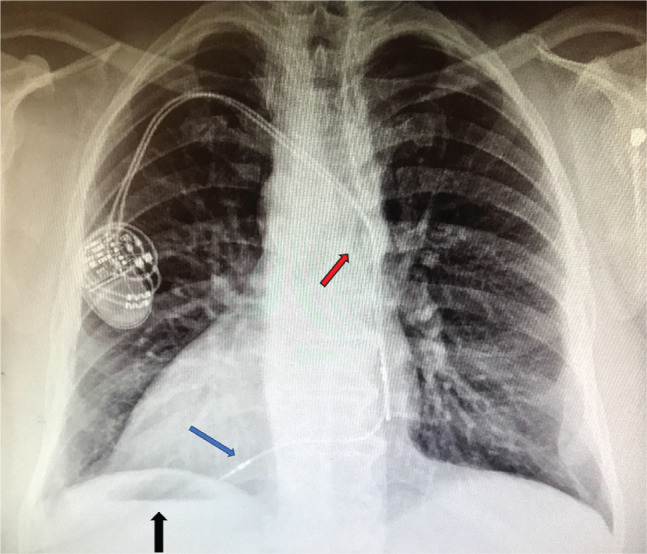
Chest X-ray showing DX-SIT. The black arrow points to a gastric bubble under the right hemidiaphragm. The red arrow points to the atrial and ventricular leads in the left-sided SVC. The blue arrow points to the ventricular lead.

A 57-year-old male with the diagnosis of DX-SIT and congestive heart failure with a left ventricular ejection fraction (LVEF) of 30% to 35% and a New York Heart Association (NYHA) functional class II designation was referred for possible CRT upgrade. The patient had received a dual-chamber pacemaker at the age of 50 years with the diagnosis of complete heart block. At the time of pacemaker implantation (seven years prior to the current presentation), his left ventricular (LV) function was normal. Ischemic heart disease was ruled out by cardiac nuclear study. Interrogation of his dual-chamber pacemaker demonstrated a sensed atrial rhythm (believed most likely to be sinus rhythm) and 100% right ventricular pacing. Therefore, the diagnosis of pacemaker-induced cardiomyopathy was made and the patient was deemed a candidate for biventricular (BIV) pacing upgrade. After discussing the alternatives, the patient agreed to a CRT pacemaker (CRT-P) device upgrade. Chest X-ray **(Figure 1)** showed dextrocardia and the presence of a gastric bubble under the right hemidiaphragm (situs inversus) as well as the location of the pacemaker generator in the right pectoral region. The course of the right atrial (RA) and right ventricular (RV) leads from the right subclavian vein through the left-sided SVC to the right-sided chamber at its mirror position of normal expected anatomy was also noted.

An electrocardiogram of the pacemaker rhythm demonstrated a sensed (believed to be sinus rhythm) P-wave, which was negative in lead I and positive in lead aVR, as well as a completely negatively paced QRS in leads V1 to V6, all of which were consistent with the patient’s diagnosis of dextrocardia. The QRS width of the RV paced rhythm was 190 ms.

**Figure 2:**
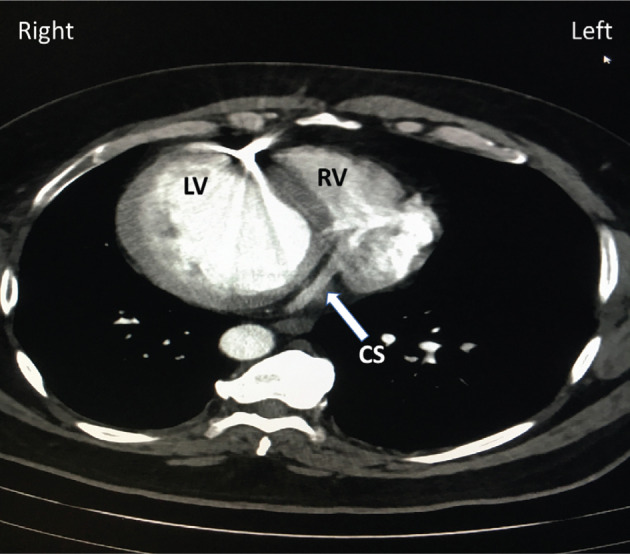
A chest CT scan demonstrating mirror imaging of normal anatomy and dextrocardia. The arrow points to the location of the CS. The right and left sides of the chest are noted. LV: left ventricle; RV: right ventricle; CS: coronary sinus.

Prior to the CRT-P upgrade procedure, we performed a chest computed tomography (CT) scan with intravenous contrast to define the preprocedural anatomy. The chest CT scan ruled out the presence of persistent right SVC. The patient had only one left-sided SV as was expected in an individual with situs inversus. The CT scan also demonstrated the location of the coronary sinus (CS) ostium and proximal coronary sinus **(Figure 2)**. Cardiac CT scan and echocardiogram ruled out the presence of congenital intracardiac defects.

### Cardiac resynchronization therapy pacemaker upgrade procedure

**Figure 3:**
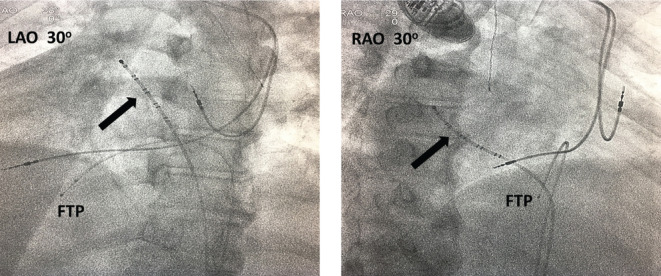
LAO and RAO projections of the CS cannulated from the left femoral venous approach. The decapolar catheter in the CS as a fluoroscopic landmark is represented by the arrow. A femoral temporary pacemaker (FTP) is noticeable as well. LAO: left anterior oblique; RAO: right anterior oblique.

A right axillary venogram was performed and demonstrated patency of the vein. In the next step of the procedure, the CS was cannulated in the 30-degree (°) right anterior oblique (RAO) fluoroscopy projection from the left femoral venous approach with an F-type deflatable Webster decapolar catheter (Biosense Webster, Diamond Bar, CA, USA). The purpose of the CS cannulation from the femoral approach was to provide a fluoroscopic landmark for CS cannulation from an axillary venous approach, thereby assessing the technical difficulty in accessing the CS due to DX-SIT **(Figure 3)**.

Fluoroscopically, to engage the CS in this patient, we used RAO projection [as the equivalent of left anterior oblique (LAO) projection in the case of normal levocardial anatomy].

**Figure 4:**
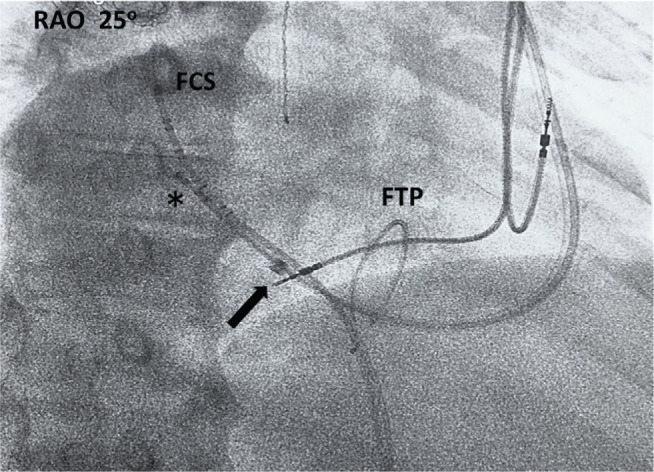
RAO projection showing advancement of the Jumbo Worley™ CSG (Merit Medical Systems, South Jordan, UT, USA), denoted by the arrow, over the quadripolar electrophysiology catheter (*) inside the CS. The femoral decapolar CS catheter (FCS) and femoral temporary pacemaker wire (FTP) are also noted.

Technically, to cannulate the CS from a femoral venous approach, a counterclockwise rotation of the deflectable catheter was required to guide the catheter from the RV to the CS in this dextrocardial anatomy (in contrast to a clockwise rotation with a similar approach in a normal levocardial anatomy). Subsequently, the pacemaker pocket was opened and a right axillary venous access attempt was made. We tried engaging the CS using a 9-French (Fr) Jumbo Worley™ coronary sinus guide (CSG) with a braided core (Merit Medical Systems, South Jordan, UT, USA) via a right axillary approach. However, it seemed that the CSG could not reach the CS ostium. Therefore, to be able to reach the CS ostium and overcome the vertical orientation of the proximal CS, we introduced a deflectable D-type quadripolar electrophysiology catheter (Biosense Webster, Diamond Bar, CA, USA) inside the Jumbo Worley™ CSG (Merit Medical Systems, South Jordan, UT, USA) after removing the braided core from the latter. The deflectable D-type electrophysiology catheter enabled us to cannulate the CS ostium and, subsequently, the Jumbo Worley™ CSG sheath (Merit Medical Systems, South Jordan, UT, USA) was advanced over the catheter into the coronary sinus. Then, finally, the catheter was removed **(Figure 4)**. Technically, a clockwise rotation of the catheter was required to allow it to fall in the coronary sinus from the right ventricle in this heart with dextrocardia approaching via the axillary vein (in contrast with the counterclockwise rotation that is required with a similar approach in a normal levocardial anatomy).

**Figure 5:**
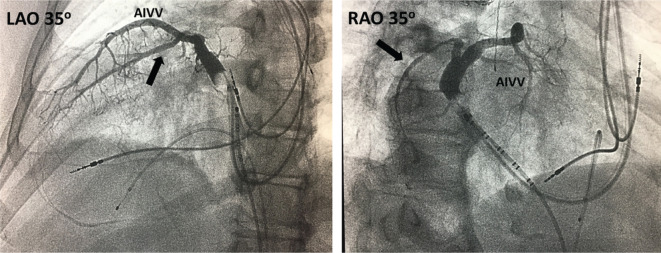
Balloon occlusion CS venography in LAO and RAO projections. The RAO projection in dextrocardia serves as the equivalent of the LAO projection in the normal heart. The optimal lateral marginal vein for the LV lead is noted by the arrow. AIVV: anterior interventricular vein; LAO: left anterior oblique; RAO: right anterior oblique.

Subsequently, a CS balloon occlusion venogram was performed in 35° LAO and 35° RAO projections **(Figure 5)**. The RAO projection showed an ideal left lateral marginal branch. At this point, we used a telescopic approach described by Jackson et al. to subselect the target lateral marginal vein.^10^ In this approach, we used a 0.014 BMW Universal Wire (Abbott Laboratories, Chicago, IL, USA); a 5-Fr hydrophilic CS VERT Worley™ vein selector (Merit Medical Systems, South Jordan, UT, USA); a 90° Attain Select™ II delivery guide (Medtronic, Minneapolis, MN, USA); and a Jumbo Worley™ CSG (Merit Medical Systems, South Jordan, UT, USA).

After the removal of the vein selector, a LV quadripolar 88-cm Medtronic 4298 lead (Medtronic, Minneapolis, MN, USA) was delivered successfully over the wire inside the lateral vein. The femoral decapolar CS catheter (CS landmark) was removed from the CS before slicing the Attain Select™ II delivery guide (Medtronic, Minneapolis, MN, USA) and removing the Jumbo Worley™ CSG (Merit Medical Systems, South Jordan, UT, USA).

**Figure 6:**
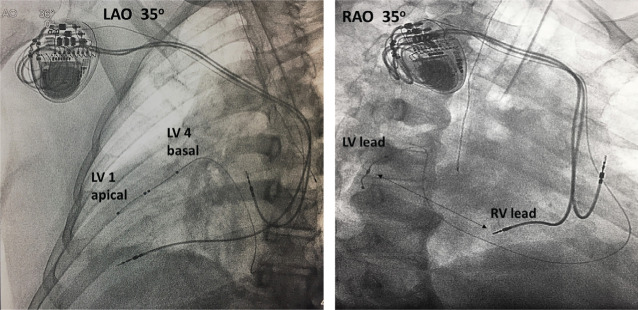
Final lead position in LAO 35° and RAO 35° projections. The LAO projection demonstrates LV4 (the most proximal LV lead pole) at the basal segment and LV1 (the most distal LV lead pole) in the apical LV segment. The RAO projection shows very lateral positioning of the LV lead with an excellent RV–LV lead separation (arrow). LAO: left anterior oblique; RAO: right anterior oblique.

**Figure 6** demonstrates the final lead positions in LAO 35° and RAO 35° projections. The RAO projection shows optimal positioning of the LV lead in a very right lateral position (mirror image of normal anatomy) with an excellent RV–LV lead separation. The LAO projection demonstrates LV4 (the most proximal pole of the LV lead) being positioned at a very basal segment of the LV, which is ideal for effective BIV pacing. LV lead pacing configuration was programmed LV4–LV1 (cathode, LV4: most proximal pole and anode, LV1: most distal pole of the lead), with an excellent capture threshold and absence of phrenic stimulation.

**Figure 7:**
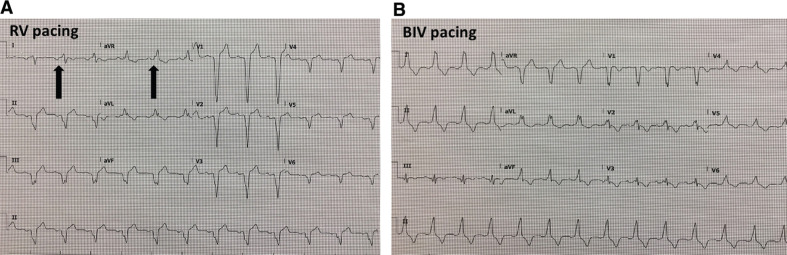
Electrocardiograms of pure RV pacing (pre-CRT) and BIV pacing (post-CRT). **A:** A before-CRT electrocardiogram demonstrating sinus rhythm with a negative P-wave in lead I and a positive P-wave in lead aVR (arrow). The RV-paced QRS is negative in V1 to V6. The QRS in lead II is negative and positive in lead aVR. The RV-paced QRS width is 190 ms. **B:** An electrocardiogram of BIV pacing (after CRT upgrade) showing sinus rhythm as described previously with the BIV-paced QRS positive in lead I as well as in V3 to V6. More importantly, the vector of the QRS is now negative in lead aVR and positive in lead II, which indicates that the vector starts at the basolateral segment of the LV, which is the optimal position considering this patient’s dextrocardial anatomy. The BIV-paced QRS width is 162 ms. RV: right ventricle; BIV: biventricular.

**Figure 7** compares the electrocardiogram before and after BIV upgrade. The electrocardiogram of BIV pacing demonstrates a QRS duration of 163 ms with a negative QRS in lead aVR and a positive QRS in lead II, indicating a favorable QRS vector for BIV pacing in this dextrocardial anatomy. Conversely, an initial negative QRS deflection in lead aVF and initial positive QRS in lead III indicates a good basolateral positioning of the LV lead and a favorable CRT response in a normal levocardial anatomy.

## Discussion

DX-SIT is a rare condition; furthermore, less than 10% of individuals with DX-SIT have associated congenital cardiac defects. Therefore, the majority of these people are increasingly surviving to adulthood and may develop coronary artery disease, heart block, and heart failure, which may require cardiac electronic device implantation. There are case reports published on the association of both congenital heart block and acquired degeneration of the conduction system with DX-SIT.^7–9^ Regardless, the exact mechanism of complete heart block in this case is still not clear.

The approach to transvenous lead implantation needs to be tailored to a patient’s individual anatomy. Therefore, it is helpful to perform imaging studies such as a CT scan or magnetic resonance imaging scan prior to the cardiac device implantation procedure in order to completely understand the patient’s cardiovascular anatomy, particularly their thoracic venous anatomy. Equally, it is important to rule out the presence of cardiac congenital defects such as intracardiac shunts by using such imaging modalities or echocardiography.

Venous return anomalies are commonly associated with DX-SIT. The expected normal SVC setup instead appears in patients with DX-SIT as only one SVC located at the left side of the spine as the mirror image of normal anatomy. DX-SIT could be associated with persistent right SVC, which may either complicate or facilitate transvenous lead implantation.^6^

This case illustrates a successful CRT-P upgrade in a patient with DX-SIT and pacemaker-induced cardiomyopathy utilizing standard tools and an unconventional technique of cannulating the CS using a deflectable electrophysiology catheter via both the femoral and axillary venous approaches.

In the absence of persistent right SVC, right axillary or subclavian venous access will make CS cannulation more straightforward^3^ (due to the mirror image of normal levocardial anatomy in which the left axillary approach is easier for the CS cannulation). If a high-energy device is desired, then a right-sided implant may provide a more effective shocking vector for a patient with DX-SIT.^3^

This case illustrates that the coronary sinus can be cannulated from a femoral approach with an electrophysiology catheter as a fluoroscopic landmark, if one anticipates difficult or abnormal anatomy for the CS cannulation from the subclavian approach. Also, we used a deflectable electrophysiology catheter inside a Jumbo Worley™ CSG (Merit Medical Systems, South Jordan, UT, USA) to cannulate the SC, which is not a typical or recommended way of utilizing the CSG system. We inserted two electrophysiology catheters inside the CS very carefully (via femoral and axillary approaches). Subsequently, the CSG was advanced into the CS over the D-type deflectable catheter. We did not observe any evidence of CS dissection on venography.

It is important to emphasize that, in dextrocardia, the RAO view provides the actual anteroposterior projection of the heart and is ideal for CS cannulation and for differentiating septal versus lateral positions. RAO projection in dextrocardial anatomy serves as the equivalent of LAO projection in the normal levocardial anatomy. Similarly, in dextrocardia, the LAO view provides the actual lateral projection of the heart and is an ideal view to differentiate basal versus apical location of the LV lead. LAO projection in dextrocardia serves as the equivalent of RAO projection in the normal levocardial anatomy.

Our other interesting observation in this case was “mirror images” as compared with normal anatomy. For example, in the CS cannulation from a left axillary approach in a normal heart, after entering the RV, a counterclockwise rotation would direct the catheter inferoposteriorly toward the CS ostium. In this patient with DX-SIT, when approaching from the right axillary vein, a clockwise rotation was required to gain access to the CS.

## Conclusion

Cardiac implantable electronic device implantation, particularly CRT device implantation, in patients with DX-SIT is possible after studying the details of their cardiac and venous anatomy. Physicians should carefully evaluate all available data on these patients in order to choose the most appropriate implantation approach, tools, and techniques.
